# Supplementary material for: Engineering microbial division of labor for plastic upcycling
Source: Nat Commun. 2023 Sep 26;14:5712. doi: 10.1038/s41467-023-40777-x (PMC10522701; doi:10.1038/s41467-023-40777-x)
Supplement: Supplementary file 1 — Supplementary Information [file 41467_2023_40777_MOESM1_ESM.pdf]

Supplementary Information

Engineering Microbial Division of Labor for Plastic Upcycling

Teng Bao[1,2,9], Yuanchao Qian[1,2,9], Yongping Xin[1,2,9], James J. Collins[3,4,5,*], and Ting Lu[1,2,6,7,8,*]

[1] Department of Bioengineering, University of Illinois Urbana-Champaign, Urbana, IL, USA

[2] Carl R. Woese Institute for Genomic Biology, University of Illinois Urbana-Champaign, Urbana, IL, USA

[3] Department of Biological Engineering and Institute for Medical Engineering & Science, Massachusetts Institute of Technology, Cambridge, MA, USA

[4] Wyss Institute for Biologically Inspired Engineering, Harvard University, Longwood, MA, USA

[5] Broad Institute of MIT and Harvard, Cambridge, MA, USA

[6] Center for Biophysics and Quantitative Biology, University of Illinois Urbana-Champaign, Urbana, IL, USA

[7] Department of Physics, University of Illinois Urbana-Champaign, Urbana, IL, USA

[8] National Center for Supercomputing Applications, Urbana, IL 61801, USA

[9] These authors contributed equally to the work.

*Corresponding authors. E-mail: luting@illinois.edu and jimjc@mit.edu

Supplementary Tables

Supplementary Table 1: Strains and plasmids used in this study.

Name	Description	Source or Reference
**Strains**		
Pseudomonas putida EM42	KT2440 derivative; Δprophage1 Δprophage4 Δprophage3 Δprophage2 ΔTn*7* Δ*endA-1* Δ*endA-2* Δ*hsdRMS* Δflagellum ΔTn*4652*	Martínez-García et al., 2014[1]
Pp00 (Pp-T_0)	EM42 derivative; contains the plasmid pBb(B5)1k-tpa, Km^R	This study
Pp01	EM42 derivative; Δ*ped*	This study
Pp02 (Pp-T)	Pp01 derivative; contains the plasmid pBb(B5)1k-tpa, Km^R	This study
Pp03 (Pp-TS)	Pp01 derivative; contains the plasmids pBb(B5)1k-tpa and pSEVA421, Sm^R, Km^R	This study
Pp04 (Pp-TP)	Pp01 derivative; contains the plasmids pBb(B5)1k-tpa and pSEVA421-phaG-alkK-phaC1-phaC2, Sm^R, Km^R	This study
Pp05	Pp01 derivative; Δ*ped,* Δ*phaZ*, Δ*fadBA* Δ*fadBAxE*	This study
Pp06 (PpΔ-TP)	Pp05 derivative; contains the plasmids pBb(B5)1k-tpa and pSEVA421-phaG-alkK-phaC1-phaC2, Sm^R, Km^R	This study
Pp07	Pp01 derivative; Δ*catRBCA*	This study
Pp08 (Pp-TC)	Pp07 derivative; contains the plasmids pBb(B5)1k-tpa and pSEVA421-aroY-EcdB, Sm^R, Km^R	This study
Pseudomonas putida M31	EM42 derivative; UV mutation, has an ability to metabolize ethylene glycol	Lab stock
PpM01	M31 derivative; Δ*gclR*	This study
PpM02 (Pp-E)	PpM01 derivative; synthetic expression cassette (P_{tac}-glcDEF) in chromosome	This study
PpM03 (Pp-TE)	PpM02 derivative; contains the plasmid pBb(B5)1k-tpa, Km^R	This study
PpM03 (Pp-ES)	PpM02 derivative; contains the plasmid pSEVA421, Sm^R	This study
PpM04 (Pp-EP)	PpM02 derivative; contains the plasmid pSEVA421-phaG-alkK-phaC1-phaC2, Sm^R	This study
PpM05 (Pp-TES)	PpM02 derivative; contains the plasmids pBb(B5)1k-tpa and pSEVA421, Sm^R, Km^R	This study
PpM06 (Pp-TEP)	PpM02 derivative; contains the plasmids pBb(B5)1k-tpa and pSEVA421-phaG-alkK-phaC1-phaC2, Sm^R, Km^R	This study
PpM06 (PpΔ-E)	PpM02 derivative; Δ*phaZ* Δ*fadBA* Δ*fadBAxE*	This study
PpM07 (PpΔ-EP)	PpM06 derivative; contains the plasmid pSEVA421-phaG-alkK-phaC1-phaC2, Sm^R	This study
PpM08 (PpΔ-TEP)	PpM06 derivative; contains the plasmids pBb(B5)1k-tpa and pSEVA421-phaG-alkK-phaC1-phaC2, Sm^R, Km^R	This study
PpM09	PpM02 derivative; Δ*catRBC* with synthetic expression cassette (P_{tac}-catA*)* in chromosome	This study
PpM10 (Pp-EM)	PpM09 derivative; contains the plasmid pSEVA421, Sm^R	This study

PpM11 (Pp-TEM)	PpM09 derivative; contains the plasmids pBb(B5)1k-tpa and pSEVA421-aroY-ecdB, Sm^R, Km^R	This study
**Plasmids**		
pBb(B5)1k-GFPuv	*E. coli* and *P. putida* shuttle vector, IPTG-inducible P_{trc} promoter expressing GFPuv, BBR1-B5 origin, Km^R	Cook et al., 2018[2]
pBb(B5)1k-tpa	Expression plasmid, constitutive expression of tpa operon (tpaAa-tpaAb-tpaC-tpaB-tpaK from *Rhodococcus jostii* RHA1) using P_{tac} promoter, Km^R	This study
pSEVA421	*E. coli* and *P. putida* shuttle vector, oriV (RK2) origin; Sm^R	Silva-Rocha et al., 2012[3]
pSEVA421-phaG-alkK-phaC1-phaC2	Expression plasmid, constitutive expression of phaG-alkK - phaC1-phaC2 using P_{EM7} promoter, Sm^R	This study
pSEVA421-aroY-ecdB	Expression plasmid, constitutive expression of aroY-ecdB using P_{tac} promoter, Sm^R	This study
pK18mobsacB	Gene knockout vector, Km^R	Schäfer et al., 1994[4]
pK18-ped	Gene knockout vector, pK18mobsacB containing the homologous arms of the *ped* DNA region of EM42, Km^R	This study
pK18-gclR	Gene knockout vector, pK18mobsacB containing the homologous arms of the *gclR* DNA region of EM42, Km^R	This study
pK18-Pro-glcDEF	Gene knockout vector, pK18mobsacB containing the homologous arms of the *glcDEF* DNA region of EM42 and P_{tac}-glcDEF cassette, Km^R	This study
pK18-phaZ	Gene knockout vector, pK18mobsacB containing the homologous arms of the *phaZ* DNA region of EM42, Km^R	This study
pK18-fadBA	Gene knockout vector, pK18mobsacB containing the homologous arms of the *fadBA* DNA region of EM42, Km^R	This study
pK18-fadBAxE	Gene knockout vector, pK18mobsacB containing the homologous arms of the *fadBAxE* DNA region of EM42, Km^R	This study
pK18-catRBC	Gene knockout vector, pK18mobsacB containing the homologous arms of the *catRBC* DNA region of EM42 and P_{tac} promoter, Km^R	This study
pK18-catRBCA	Gene knockout vector, pK18mobsacB containing the homologous arms of the *catRBCA* DNA region of EM42, Km^R	This study

Supplementary Table 2: Sequence information for genes, promoters, and insertion fragments.

Primer Name	Primer sequence	Description
**For *ped* gene deletion plasmid construction**		
PK18-F	CCGGATGAATGTCAGCTACT	To clone the 5100 bp DNA fragment from the pK18mobsacB.
PK18-R	GCGGTAATACGGTTATCCAC	
ped-UF	TACAGGCCCCACCGCGTCGCGGCCTTC	To clone the 1481 bp upstream DNA fragment of *ped* gene from the *P. putida* EM42 chromosome.
ped-UR	AGTAGCTGACATTCATCCGGTCTCGCGCAGCGCAATATCAGTAGGG	
ped-DF	GTGGATAACCGTATTACCGCGCTGGTGCACAACTTGTATCCG	To clone the 1292 bp downstream DNA fragment of *ped* gene from the *P. putida* EM42 chromosome.
ped-DR	GACGCGGTGGGGCCTGTAGGCGC	
V-ped-F	TGCGGGTGGCGGGGTGA	To verify the *ped* gene deletion strain, wild strain yields 10002 bp, the Δ*ped* yields 409 bp.
V-ped-R	TTAATGCACGGGGGCGGACTG	
**For *gclR* gene deletion plasmid construction**		
pk18-F1	TGGCGCACATCCATAAGCTTGGCACTGGCCGTCGTTTTACAA	To clone the 5724 bp DNA fragment from the pK18mobsacB.
pk18-R1	CAGATAACCCTTCTGGGATCCCCGGGTACCGAGCTCGAATTCGTAA	
gclR-UF	CACGCAGGCAAACGAGGGCTCTCGAGGCACGAAGAGAAAGTATGG	To clone the 1462 bp upstream DNA fragment of *gclR* gene from the *P. putida* EM42 chromosome.
gclR-UR	GCCAGTGCCAAGCTTATGGATGTGCGCCATGCTGCCCATCATC	
gclR-DF	GGTACCCGGGGATCCCAGAAGGGTTATCTGGACGTCTACGATCACTTCGAGCTGC	To clone the 1431 bp downstream DNA fragment of *gclR* gene from the *P. putida* EM42 chromosome.
gclR-DR	GTGCCTCGAGAGCCCTCGTTTGCCTGCGTGATCGAGGGGCTG	
V-gclR-F	ATCACCTGGGCGATCACATAAGGCAG	To verify the ped gene deletion strain, wild strain yields 1646 bp, the Δ*gclR* yields 409 bp.
V-gclR-R	CCATGATGTTGGGGTTGACGAAGATGATGTAGG	
**For P_{tac}-glcDEF integration plasmid construction**		
PK18-F	CCGGATGAATGTCAGCTACT	To clone the 5100 bp DNA fragment from the pK18mobsacB.
PK18-R	GCGGTAATACGGTTATCCAC	
glcDEF-UF	ATGAATATCCTGTACGACGAACGCGTCG	To clone the 1520 bp upstream DNA fragment of *glcDEF* operon from the *P. putida* EM42 chromosome.
glcDEF-UR	AGTAGCTGACATTCATCCGGTCAGAAGCGCTCCAGCTCGGGGAAGG	
glcDEF-DF	GTGGATAACCGTATTACCGCCCGCTTCGCTGTATTCGACCTTCAAGG	To clone the 1254 bp downstream DNA fragment of *glcDEF* operon from the *P. putida* EM42 chromosome.
glcDEF-DR	CGGCTCACTCGCAACGGTTTTTGTTGTTGTTGG	

V-glcDEF-F	ATATCGCCAATCTAGCCAGACAGAACCG	To verify the constitutively expression *glcDEF* strain, wild strain yields 790 bp, the engineered strain yields 897 bp.
V-glcDEF-R	TGAACAATGCCAGCAGGTCGAAGC	
P_{tac}-RBS	ACAACAACAAAAACCGTTGCGAGTGAGCCGCGCAAAAAACCGCACCCAGGTGCGGTTTTTTGAATTCGAGCTGTTGACAATTAATCATCGGCTCGTATAATGTGTCAGACTCAATAATAATAATAAGGAGGTATCGAATGAATATCCTGTACGACGAACGCGTCG	Synthesized DNA fragment of strong constitutive promoter (P_{tac}) and an artificial RBS. Gibson overlaps are underlined.
**For *catRBC* gene deletion plasmid construction**		
catRBC-UF	ACCTGACCTCCATAGACACACCATGCCCACAGGGG	To clone the 1234 bp upstream DNA fragment of *catRBC* gene from the *P. putida* EM42 chromosome.
catRBC-UR	GTAGCTGACATTCATCCGGCAAGGCGCAGGAAAAAGGGTTGC	
catRBC-ptac-F	AATTTTCACGGTCATATGATACCCTCGTGTGTGAGTTAATC	To clone the 149 bp P_{tac} promoter.
catRBC-ptac-R	GTGGGCATGGTGTGTCTATGGAGGTCAGGTATGATTACTATTGA	
catRBC-DF	GTGGATAACCGTATTACCGCAGGAAGTTGAGCAAGTCCGG	To clone the 1254 bp downstream DNA fragment of *catRBCA* operon from the *P. putida* EM42 chromosome.
catRBC-DR	ACGAGGGTATCATATGACCGTGAAAATTTCCCACACTGC	
V-catRBC-F	GTGTCTTGCAGCACGCGCAG	To verify the *catRBC* gene deletion strain, wild strain yields 2853 bp, the Δ*catRBC* yields 487 bp.
V-catRBC-R	CGCATGCACCGCAAGAACCTGTAC	
**For *catRBCA* gene deletion plasmid construction**		
catRBCA-UF	GACATAACCTCGAACACACACCATGCCCACAGGGG	To clone the 1234 bp upstream DNA fragment of *catRBCA* gene from the *P. putida* EM42 chromosome.
catRBCA-UR	GTAGCTGACATTCATCCGGCAAGGCGCAGGAAAAAGGGTTGC	
catRBCA-DF	GTGGATAACCGTATTACCGCAATCGAGAAACTGCGAAAGCTTGGC	To clone the 1270 bp downstream DNA fragment of *catRBCA* operon from the *P. putida* EM42 chromosome.
catRBCA-DR	GTGGGCATGGTGTGTGTTCGAGGTTATGTCACTGTGATTTTGC	
V-catRBCA-F	GGTAGAGATTGCGGATCAGCGACG	To verify the *catRBCA* gene deletion strain, wild strain yields 3847 bp, the Δ*catRBCA* yields 426 bp.
V-catRBC-R	CGCATGCACCGCAAGAACCTGTAC	
**For *phaZ* gene deletion plasmid construction**		
phaZ-UF	TTTCCTGCGTTATCCCCTGATTCTGTGGATAGCAGACCTTCATCATCAGC	To clone the 1048 bp upstream DNA fragment of *phaZ* gene from the *P. putida* EM42 chromosome.
phaZ-UR	TGTCAGGCCGCAGCTGTTGCACGTGACTCTTGGGTG	

phaZ-DF	CACCCAAGAGTCACGTGCAACAGCT GCGGCCTGACA	To clone the 1048 bp downstream DNA fragment of *phaZ* gene from the *P. putida* EM42 chromosome.
phaZ-DR	CAGATAGCCCAGTAGCTGACATTCAT CCGGCCGGCGCAATTGCTTCTT	
V-phaZ-F	TCTTTAACGGCATCGGC	To verify the *phaZ* gene deletion strain, wild strain yields 308 bp, the Δ*phaZ* yields 0 bp.
V-phaZ-R	TGGCACCATTACCGCA	
**For *fadBAxE* gene deletion plasmid construction**		
fadBAxE-UF	TTTCCTGCGTTATCCCCTGATTCTGTG GATAGCTGGGTATCACCAACCTG	To clone the 893 bp upstream DNA fragment of *fadBAxE* operon from the *P. putida* EM42 chromosome.
fadBAxE-UR	ATTTGCTTCCACTGGAGTACTTTCCTT TCAGACGCT	
fadBAxE-DF	CTGAAAGGAAAGTACTCCAGTGGAA GCAAATTCGCA	To clone the 821 bp downstream DNA fragment of *fadBAxE* operon from the *P. putida* EM42 chromosome.
fadBAxE-DR	CAGATAGCCCAGTAGCTGACATTCAT CCGGCACATCGACTCGGCTATTCA	
V-fadBAxE-F	GACCTCAAGAGCCTGACTGC	To verify *fadBAxE* operon deletion, wild strain yields 966 bp, the Δ*fadBAxE* yields 0 bp.
V-fadBAxE-R	GCCGTGGATATTGACCTTGT	
**For *fadBA* gene deletion plasmid construction**		
fadBA-UF	TTTCCTGCGTTATCCCCTGATTCTGTG GATATGTCCTTCATGCGCGGC	To clone the 1043 bp upstream DNA fragment of *fadBA* operon from the *P. putida* EM42 chromosome.
fadBA-UR	TCCCGCTACGCGCAACTGATCTCCAC GATATGGAAG	
fadBA-DF	TATCGTGGAGATCAGTTGCGCGTAGC GGGACAGCAG	To clone the 1048 bp downstream DNA fragment of *fadBA* operon from the *P. putida* EM42 chromosome.
fadBA-DR	CAGATAGCCCAGTAGCTGACATTCAT CCGGGGCCCACAGCAGTGGCGA	
V-fadBA-F	GTCTTCGATGCCACCGTG	To verify *fadBA* operon deletion, wild strain yields 712 bp, the Δ*fadBA* yields 0 bp.
V-fadBA-R	GTTACCGGTCATGATCGCC	
**For pBb(B5)1k-tpa plasmid construction**		
Ptac-TPA-F	GGCAATTCCGACGTCCTATGGAGGTC AGGTATGATTACTATTGA	To clone the 143 bp P_{tac} promoter.
ptac-TPA-R	GCTTTCCATATGATACCCTCGTGT	
TPA-F	ACACGAGGGTATCATATGGAAAGCAG CGTTGTCGACACGGT	To clone the 5218 bp *tpa* operon from *Rhodococcus jostii* RHA1.
TPA-R	CGAGTTTGGATCCCTACTTGCGGGCG AGCGAATGACTTT	
BR5-F	TCGCCCGCAAGTAGGGATCCAAACTC GAGTAAGGAT	To clone the 2517 bp BR5 origin from pBb(B5)1k-GFPuv plasmid.
BR5-R	CTGACCTCCATAGGACGTCGGAATTG CCAGC	

For pSEVA421-aroY-ectB plasmid construction		
pSEVA-F	ACTAGTCTTGGACTCCTGTTGATAG	To clone the 3865 bp RK2 origin from pSEVA421 plasmid.
Ptac-R	ATGATACCCTCGTGTGTGAGTTAAT	
aroY-frag1	ATTAACTCACACACGAGGGTATCATAT<br>GCAGAACCCGATCAACGACCTGCGCT<br>CCGCGATCGCGCTGCTGCAACGCCAT<br>CCGGGTCACTACATCGAAACCGACCA<br>CCCGGTCGACCCGAACGCCGAACTG<br>GCCGGTGTGTACCGCCACATCGGTGC<br>GGGTGGCACCGTGAAACGTCCGACC<br>CGCACCGGTCCAGCCATGATGTTCAA<br>CAGCGTGAAGGGCTACCCAGGCAGC<br>CGCATCCTGGTGGGCATGCACGCCAG<br>CCGTGAACGTGCCGCCCTGCTGCTGG<br>GCTGCGTGCCAAGCAAACTGGCGCA<br>GCACGTGGGCCAGGCCGTGAAGAAC<br>CCGGTGGCCCCAGTGGTGGTGCCAG<br>CCAGCCAAGCCCCATGCCAAGAACA<br>GGTGTTCTACGCCGACGACCCGGACT<br>TCGACCTGCGCAAGCTGCTGCCAGCC<br>CCAACCAACACCCCGATCGATGCCGG<br>TCCGTTCTTCTGCCTGGGCCTGGTGC<br>TGGCGAGCGACCCGGAAGATACCAG<br>CCTGACCGACGTGACCATCCACCGCC<br>TGTGCGTGCAAGAGCGCGACGAGCT<br>GAGCATGTTCCTGGCCGCCGGTCGCC<br>ACATCGAGGTGTTCCGCAAGAAGGC<br>CGAAGCCGCCGGTAAGCCGCTGCCG<br>GTGACCATCAACATGGGCCTGGACCC<br>AGCCATCTACATCGGTGCCTGCTTCG<br>AAGCGCCAACCACCCCGTTCGGCTAC<br>AACGAGCTGGGTGTGGCC	Synthesized DNA fragment 1 of *aroY*. Gibson overlaps are underlined.
aroY-frag2	ACAACGAGCTGGGTGTGGCCGGTGC<br>CCTGCGTCAGCAACCGGTGGAACTG<br>GTGCAGGGCGTGGCCGTGAAAGAG<br>AAGGCGATCGCGCGTGCCGAGATCAT<br>CATCGAGGGCGAACTGCTGCCAGGC<br>GTGCGCGTGCGCGAAGATCAGCACA<br>CCAACACCGGTCACGCCATGCCGGA<br>ATTCCCAGGCTACTGCGGTGAGGCCA<br>ACCCGAGCCTGCCGGTGATCAAGGT<br>GAAGGCCGTGACCATGCGCAACCAC<br>GCCATCCTGCAGACCCTGGTGGGTCC<br>GGGTGAGGAACACACCACCCTGGCG<br>GGTCTGCCGACCGAAGCCAGCATCC<br>GCAACGCCGTGGAAGAGGCGATCCC<br>AGGCTTCCTGCAGAACGTGTACGCCC<br>ACACCGCCGGTGGCGGTAAGTTCCT	Synthesized DNA fragment 2 of *aroY*. Gibson overlaps are underlined.

	GGGCATCCTGCAGGTCAAGAAGCGC<br>CAGCCGAGCGACGAAGGCCGTCAGG<br>GCCAAGCCGCCCTGATCGCCCTGGCC<br>ACCTACAGCGAGCTGAAGAACATCAT<br>CCTGGTGGACGAGGACGTGGACATC<br>TTCGACAGCGACGACATCCTGTGGGC<br>CATGACCACCCGCATGCAGGGCGAC<br>GTGAGCATCACCACCCTGCCAGGCAT<br>CCGTGGCCATCAGCTGGACCCGAGC<br>CAGAGCCCAGACTACAGCACCAGCAT<br>CCGTGGCAACGGCATCAGCTGCAAG<br>ACCATCTTCGACTGCACCGTGCCGTG<br>GGCCCTGAAAGCCCGTTTCGAGCGT<br>GCCCCATTCATGGAAGTGGACCCGAC<br>CCCGTGGGCCCCAGAGCTGTTCAGC<br>GACAAGAAGTGAGGAGGTTCGAATA<br>TGCGCCT	
ecdB-frag	CAAGAAGTGAGGAGGTTCGAATATG<br>CGCCTGATCGTGGGCATGACCGGTGC<br>GACCGGTGCGCCACTGGGCGTGGCC<br>CTGCTGCAGGCCCTGCGTGATATGCC<br>GGAAGTGGAAACCCACCTGGTGATG<br>AGCAAGTGGGCCAAGACCACCATCG<br>AGCTGGAAACCCCGTACACCGCCCA<br>GGACGTGGCCGCCCTGGCCGATGTG<br>GTGCATAGCCCAGCCGATCAAGCCGC<br>CACCATCAGCAGCGGCAGCTTCCGCA<br>CCGACGGCATGATCGTGATCCCGTGC<br>AGCATGAAAACCCTGGCCGGTATCCG<br>TGCCGGTTACGCCGAAGGCCTGGTG<br>GGTCGTGCCGCCGACGTGGTGCTGA<br>AAGAGGGTCGCAAGCTGGTCCTGGT<br>GCCACGCGAAACCCCACTGAGCACC<br>ATCCACCTGGAAAACATGCTGGCCCT<br>GAGCCGCATGGGCGTGGCCATGGTC<br>CCACCGATGCCAGCCTACTACAACCA<br>CCCGCAGACCGCCGACGACATCACCC<br>AGCACATCGTGACCCGTGTGCTGGAC<br>CAGTTCGGCCTGGAACACAAGAAAG<br>CCCGTCGCTGGAACGGCCTGCAAGC<br>CGCCAAGCACTTCAGCCAAGAGAAC<br>AACGACGGCATCTGAACTAGTCTTGG<br>ACTCCTGTTGATAG	Synthesized DNA fragment of *ecdB*. Gibson overlaps are underlined.
**For pSEVA421-phaG-alkK-phaC1-phaC2 plasmid construction**		
EM7-F	CCTACGTGCTGACCCGATGAGCGTCG<br>TGACTGGGAAAACC	To clone the 3857 bp RK2 origin from pSEVA421 plasmid.
EM7-R	ATTTCTGGCCTCATGGTATATTCCTCTT<br>GCTAGAATTCGC	

phaG-F	AGCAAGAGGAATATACCATGAGGCCAGAAATCGCTGTACT	To clone the 924 bp *phaG* gene from the *P. putida* EM42 chromosome.
phaG-R	TCCCTTTTCTGAAAAGCGGTCAGATGGCAAATGCATGCTG	
alkK-F	GCATGCATTTGCCATCTGACCGCTTTTCAGAAAAGGGATC	To clone the 1745 bp *alkK* gene from the *P. putida* EM42 chromosome.
alkK-R	GACGCTCCGTTGTCCTGAGTTACAACGTGGAAAGGAACGC	
phaC1-F	TTCCTTTCCACGTTGTAACTCAGGACAACGGAGCGTCGTA	To clone the 1739 bp *phaC1* gene from the *P. putida* EM42 chromosome.
phaC1-R	ACTCCCTCGTCTGATCCATCAACGCTCGTGAACGTAGGTG	
phaC2-F	TACGTTCACGAGCGTTGATGGATCAGACGAGGGAGTGTTG	To clone the 1744 bp *phaC2* gene from the *P. putida* EM42 chromosome.
phaC2-R	GTTTTCCCAGTCACGACGCTCATCGGGTCAGCACGTAGGT	

Supplementary Table 3: Comparison of microbial mcl-PHA production from PET-derived substrates.

Substrate	Strain	Cultivation mode	mcl-PHA titer (mg/L)	mcl-PHA yield ($mg/g_{substrate}$)	mcl-PHA yield (mg/g_{CDW})	Ref.
Pure EG	*P. putida* KT2440	Batch Flask	372 [a]	60	321.9	Franden et al., 2018[5]
	Pseudomonas sp. MPC6	Batch Flask	210	NA	155	Orellana-Saez et al., 2019[6]
Pure TPA	*P. umsongensis* GO16	Fed-batch Bioreactor	2349 [a]	14 [a]	27.0 [a]	Kenny et al., 2012[7]
Pure TPA and EG	*P. umsongensis* GO16	Batch Bioreactor	130	11.95 [a,b]	90	Narancic et al., 2021[8]
Hydrolyzed PET (TPA) [c]	*P. putida* GO19	Batch Flask	~207 [a]	50 [a]	18.8 [a]	Kenny et al., 2008[9]
	P. frederiksbergensis G023	Batch Flask	~205 [a]	50 [a]	18.7 [a]	Kenny et al., 2008[9]
	P. umsongensis GO16	Fed-batch Bioreactor	~210 [a]	50 [a]	21.0 [a]	Kenny et al., 2008[9]
Hydrolyzed PET (TPA and EG)	*P. umsongensis* GO16	Batch Bioreactor	150 [a]	14 [b]	7.0	Tiso et al., 2021[10]
	P. putida consortium	Batch Flask	296.46	21.33	10.16	This study
		Fed-batch Flask	637.30	22.57	11.60	

a: Estimated from reference. b: EG did not reach complete depletion. c: EG was present in hydrolyzed PET but was not uitilized as a substrate. NA: Not available.

Supplementary Table 4: Comparison of MA production from various substrates.

Strain	Substrate	Cultivation mode	cis-cis MA titer (g/L)	cis-cis MA yield (mol/mol)	Ref.
P. putida KT2440	Glucose	Fed-batch Bioreactor	22.0	0.36	Bentley, G.J., et al., 2020[11]
	Catechol	Fed-batch Bioreactor (Glucose as an additional growth substrate)	64.2	~1.00	Kohlstedt, M., et al.,2018[12]
	Hydrolyzed PET (TPA and EG) [a]	Batch Flask (Glucose as an additional growth substrate)	4.59	1.01 [b]	Liu, P., et al., 2022[13]
P. putida EM42	Catechol	Fed-batch Bioreactor (Glucose as an additional growth substrate)	73.8	NA	Kohlstedt, M., et al., 2022[14]
E. coli MA-1	Hydrolyzed PET (TPA) [c]	Whole-cell catalyst	0.38	0.85	Kim, H.T., et al., 2019[15]
P. putida consortium	Hydrolyzed PET (TPA and EG)	Batch Flask (EG as an additional growth substrate)	4.73	0.64	This study

a: EG did not reach complete depletion. b: Probably due to the experimental error. c: EG was removed from the hydrolyzed PET solution prior to fermentation.NA: Not available.

Supplementary Figures

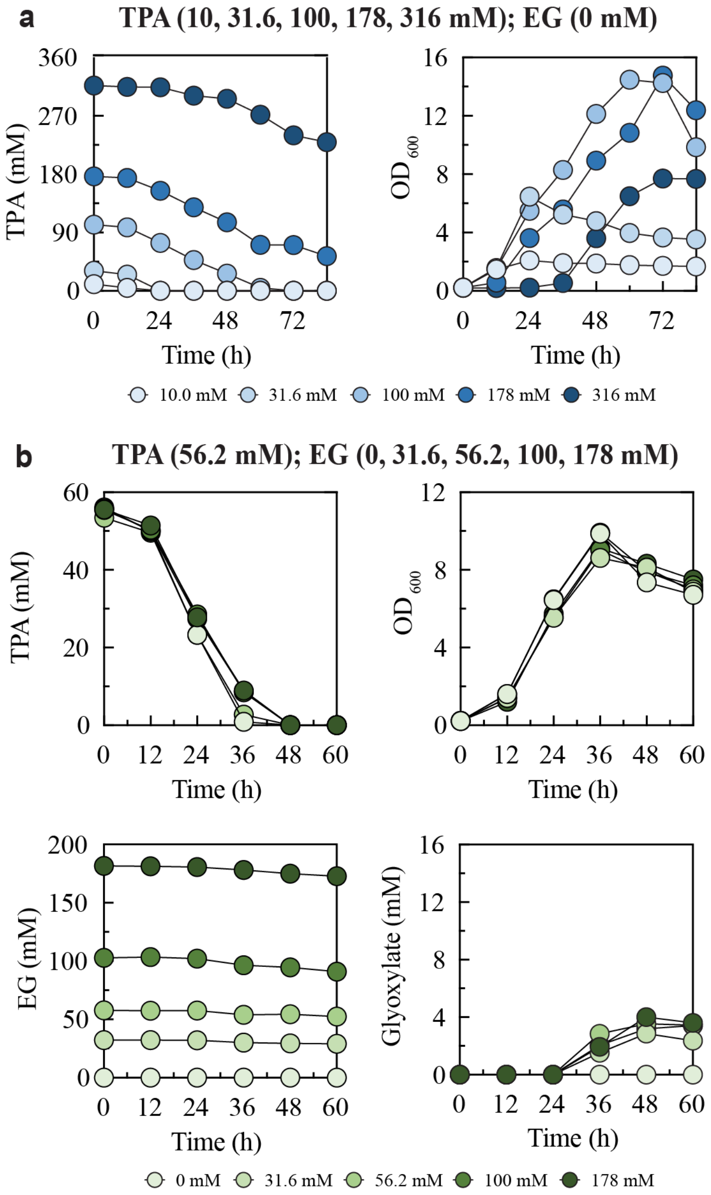

Supplementary Figure 1: TPA assimilation by the Pp-T strain under various EG levels. a, Temporal profiles of TPA assimilation (left) and cell growth (right) of Pp-T in the presence of TPA only. Initial TPA concentrations tested include 10, 31.6, 100, 178 and 316 mM. **b**, Temporal profiles of TPA assimilation (top left), cell growth (top right), EG assimilation (bottom left) and glyoxylate accumulation (bottom right) of Pp-T under 56.2 mM TPA mixed with varied EG levels (0, 31.6, 56.2, 100 and 178 mM). Experimental data are presented as mean values with standard deviations from three independent experiments. Source data are provided as a Source Data file.

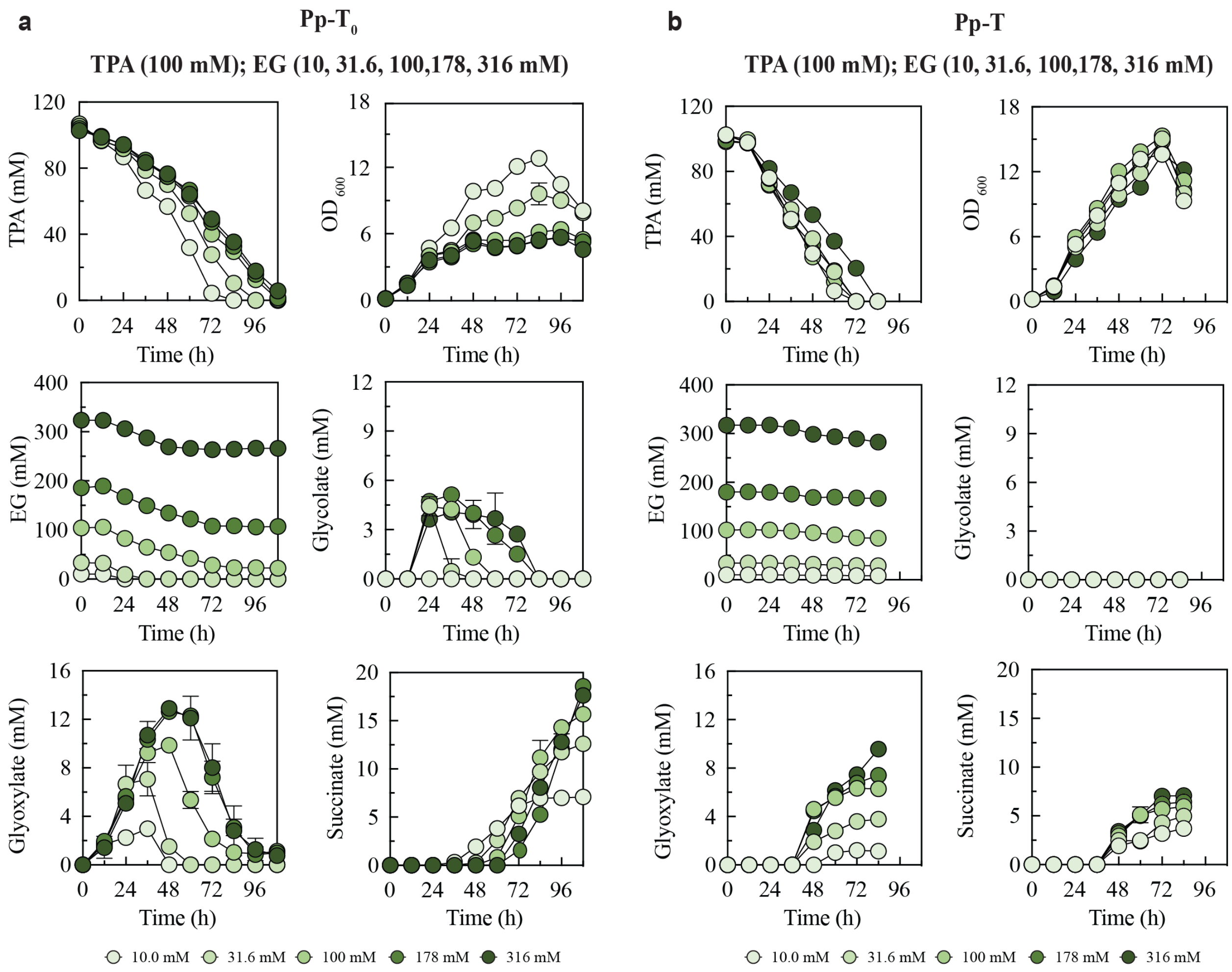

Supplementary Figure 2: Comparison of metabolite concentrations and cell growth TPA-utilizing strains Pp-T$_0$ and Pp-T. a,b, Temporal profiles of TPA assimilation (top left), cell growth (top right), EG assimilation (middle left), glycolate accumulation (middle right), glyoxylate accumulation (bottom left), and succinate accumulation (bottom right) of Pp-T$_0$ (**a**) and Pp-T (**b**). In both panels, a fixed 100 mM of TPA but varied EG concentrations (10, 31.6, 100, 178 and 316 mM) were used as different initial conditions. Experimental data are presented as mean values with standard deviations from three independent experiments. Source data are provided as a Source Data file.

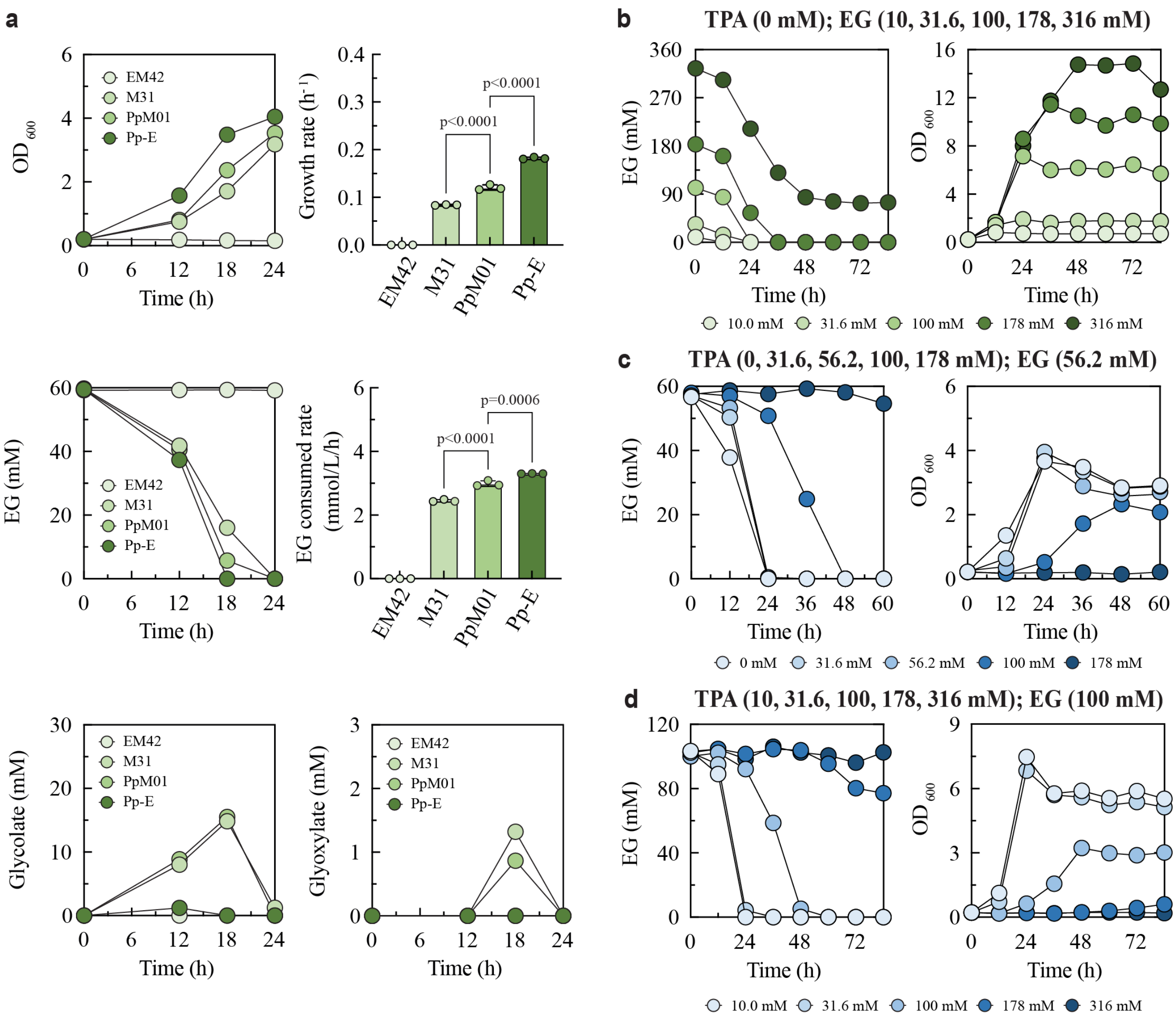

Supplementary Figure 3: EG assimilation by Pp-E and its parental strains under various TPA levels. a, Temporal profiles of cell growth (top left), growth rate (top right), EG assimilation (middle left), EG consumed rate (middle right), glycolate accumulation (bottom left) and glyoxylate accumulation (bottom right) of *P. putida* EM42, M31, PpM01 and Pp-E (PpM02) in the presence of 56 mM EG only. **b**, Temporal profiles of EG assimilation (left) and cell growth (right) of Pp-E in the presence of EG only. Initial EG concentrations tested include 10, 31.6, 100, 178 and 316 mM. **c,d**, Temporal profiles of EG assimilation (left) and cell growth (right) of Pp-E for different initial TPA and EG mixtures. Panel **c** corresponds to 56.2 mM of EG and varied initial TPA concentrations (0, 31.6, 56.2, 100 and 178 mM). Panel **d** corresponds to 100 mM of initial EG level but varied initial TPA concentrations (10, 31.6, 100, 178 and 316 mM). In panel **a**, the results

of growth rate and EG consumed rate were compared using one-way ANOVA (one-sided) with Dunnett's multiple comparison test. Experimental data are presented as mean values with standard deviations from three independent experiments. Source data are provided as a Source Data file.

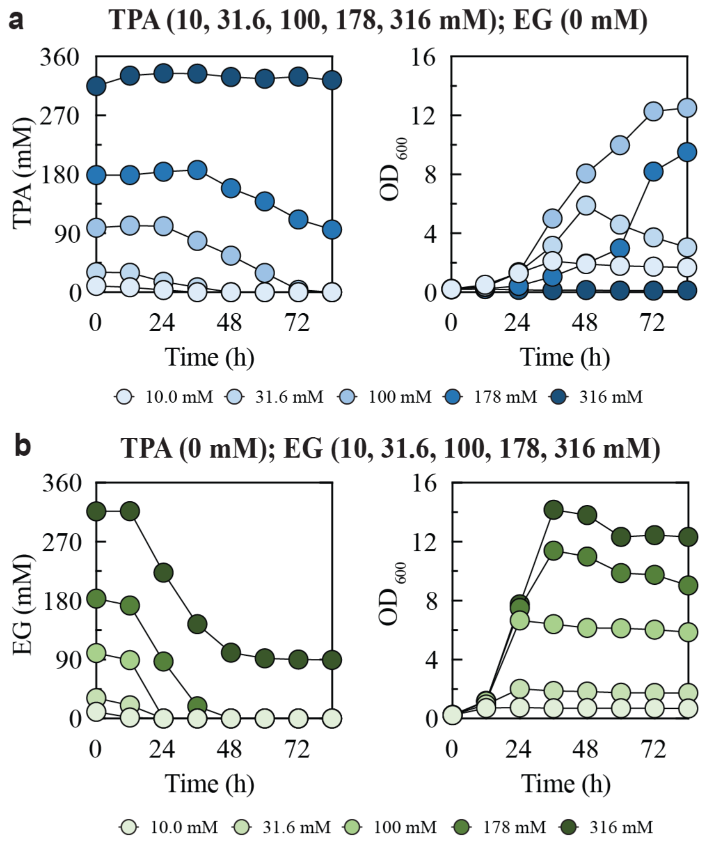

Supplementary Figure 4: TPA and EG co-consumption by the Pp-TE strain. a, Temporal profiles of TPA assimilation (left) and cell growth (right) of Pp-TE in the presence of TPA only. Initial TPA concentrations tested include 10, 31.6, 100, 178 and 316 mM. **b**, Temporal profiles of the EG assimilation (left) and cell growth (right) of Pp-TE in the presence of EG only. Initial EG concentrations tested include 10, 31.6, 100, 178 and 316 mM. Experimental data are presented as mean values with standard deviations from three independent experiments. Source data are provided as a Source Data file.

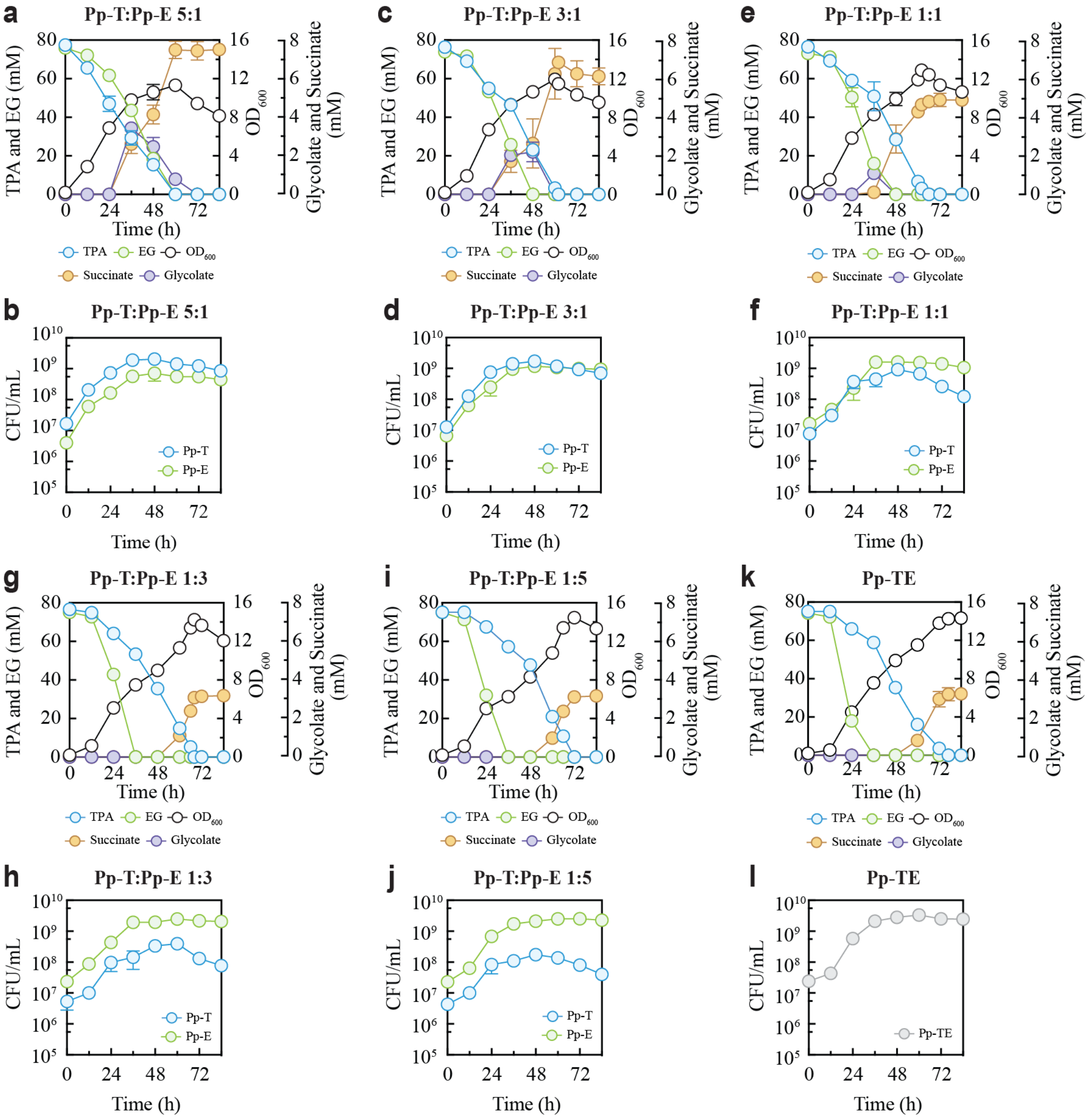

Supplementary Figure 5: Temporal profiles of metabolite concentrations and cell growth of the T-E consortium with different initial population ratios. a-j, Temporal profiles of TPA, EG, OD_{600}, succinate, glycolate, and Pp-T and Pp-E populations (CFU) of the T-E consortium. Different initial Pp-T:Pp-E ratios include 5:1 (**a,b**), 3:1 (**c,d**), 1:1 (**e,f**), 1:3 (**g,h**), and 1:5 (**i,j**). **k,l**, Temporal profiles of TPA, EG, OD_{600}, succinate and glycolate (**k**) and temporal population dynamics (**l**) of the single strain Pp-TE. The initial concentrations of TPA and EG were both 75 mM. Experimental data are presented as mean values with standard deviations from three independent experiments. Source data are provided as a Source Data file.

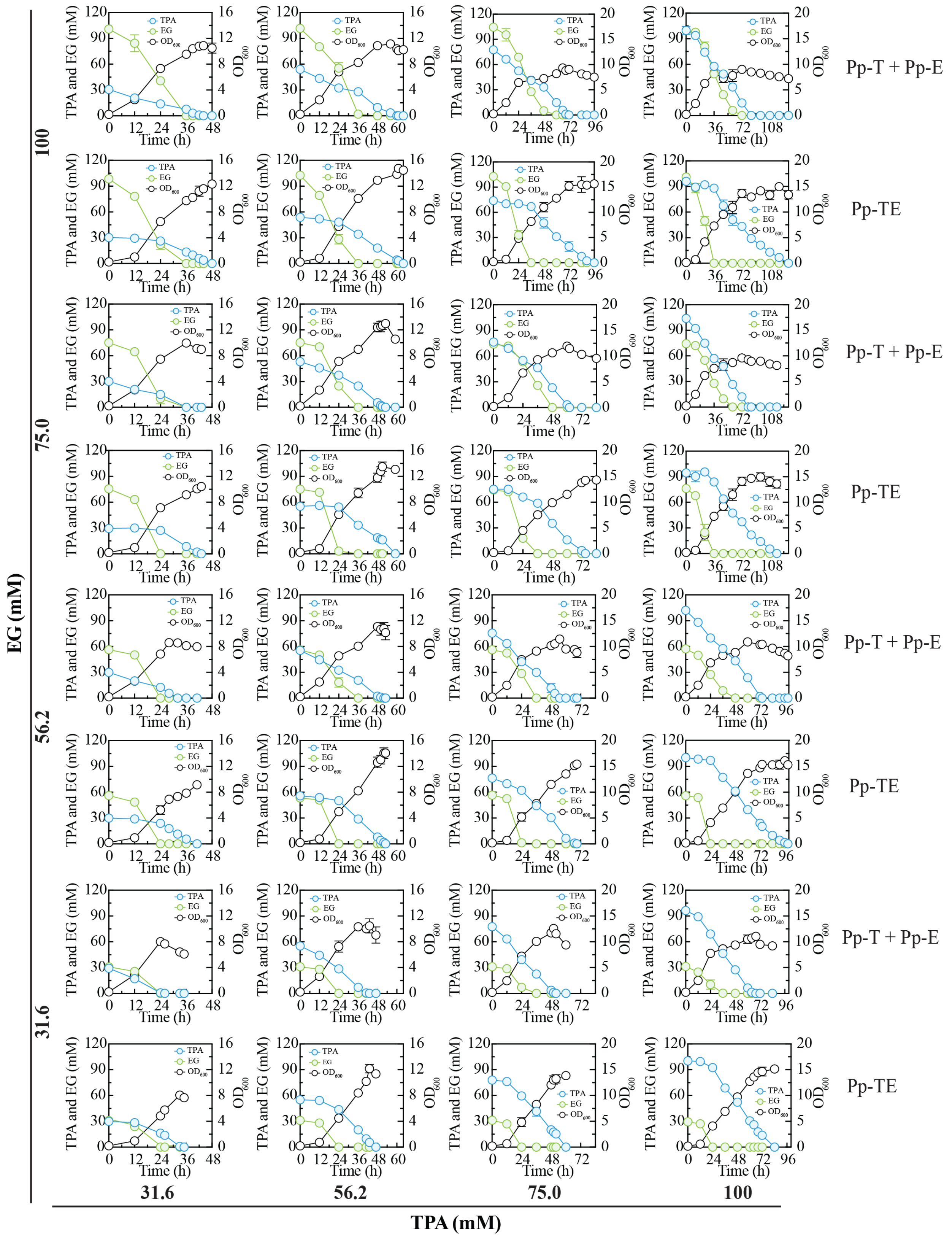

Supplementary Figure 6: Temporal profiles of substrate utilization and cell growth of the T-E consortium and Pp-TE under various TPA and EG mixtures. A total of 32 fermentations were

carried out to systematically compare the substrate degradation rates of the T-E consortium and Pp-TE under 16 substrate conditions. A 3:1 inoculation ratio was used for Pp-T and Pp-E. Experimental data are presented as mean values with standard deviations from three independent experiments. Source data are provided as a Source Data file.

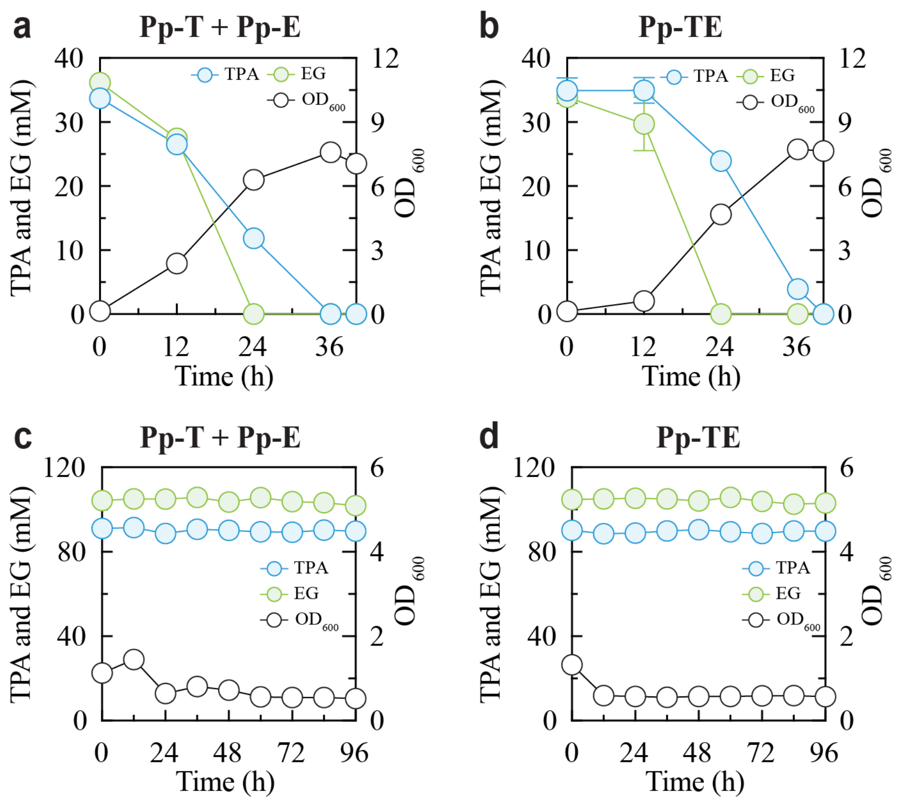

Supplementary Figure 7: Fermentation of PET hydrolysate by various strains. a,**b**, Temporal fermentation profiles of the T-E consortium (**a**) and Pp-TE (**b**) with 31.6 mM of initial PET hydrolysate. **c**,**d**, Temporal fermentation profiles of the T-E consortium (**c**) and Pp-TE (**d**) with 100 mM of initial PET hydrolysate. The measured TPA and EG concentrations deviate slightly from 100 mM due to the minor TPA precipitation at a high concentration. Experimental data are presented as mean values with standard deviations from three independent experiments. Source data are provided as a Source Data file.

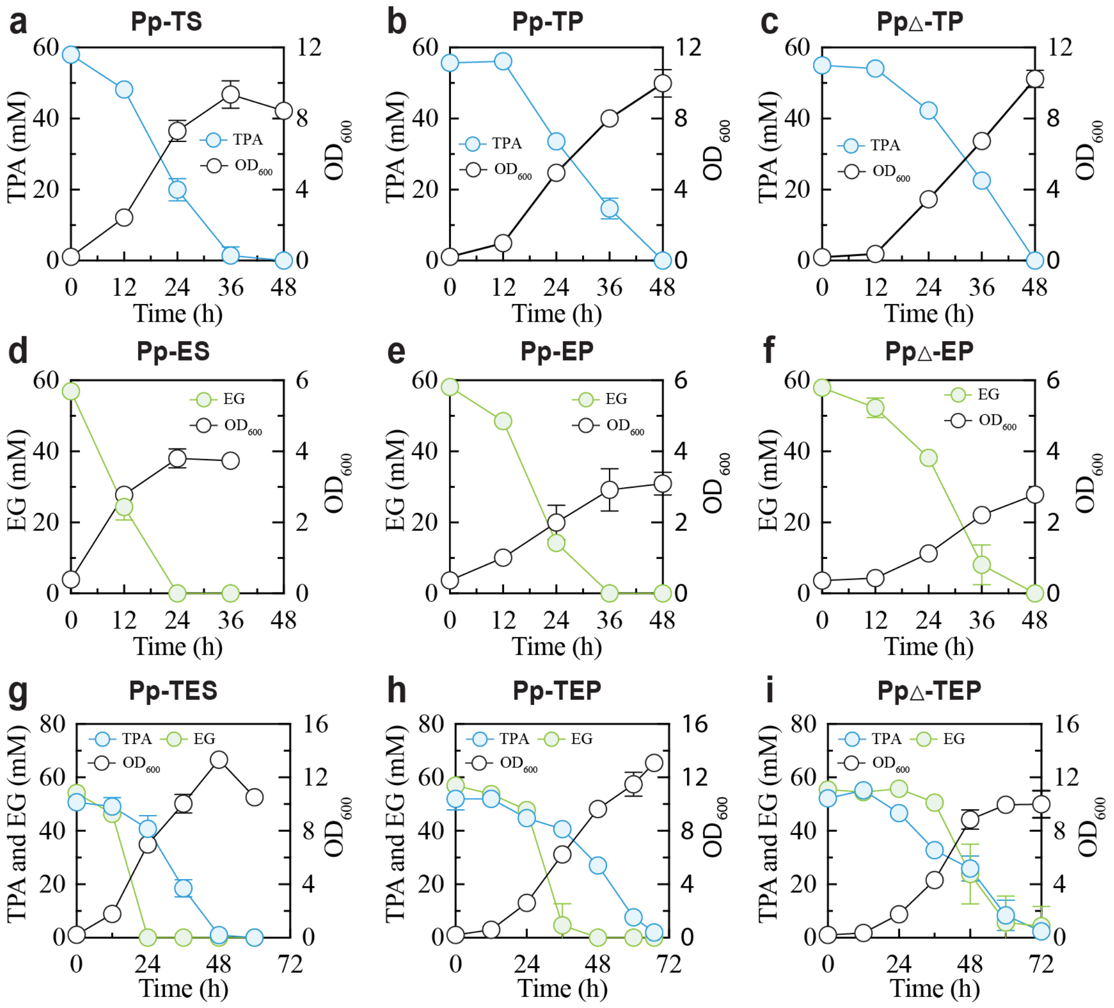

j

	Yield (mg_{PHA}/g_{CDW})	Yield (mg_{PHA}/g_{TPA})		Yield (mg_{PHA}/g_{CDW})	Yield (mg_{PHA}/g_{EG})		Yield (mg_{PHA}/g_{CDW})	Yield (mg_{PHA}/g_{TPA+EG})
**Pp-TS**	48.12±2.90	9.72±0.67	**Pp-ES**	2.56±0.17	0.96±0.06	**Pp-TES**	98.00±9.53	20.95±1.40
**Pp-TP**	64.55±4.32	14.01±1.37	**Pp-EP**	20.79±1.63	8.69±0.65	**Pp-TEP**	127.76±7.13	28.01±0.82
**Pp△-TP**	63.35±0.44	12.20±0.57	**Pp△-EP**	22.28±1.64	11.85±2.80	**Pp△-TEP**	102.05±3.05	20.25±2.67

Supplementary Figure 8: Pure TPA and EG utilization by various mcl-PHA production strains under nitrogen-surplus conditions. a-**c**, Substrate degradation and cell growth during TPA fermentation by the strains Pp-TS (**a**), Pp-TP (**b**) and Pp∆-TP (**c**), respectively. The initial TPA concentration was 56.2 mM. **d**-**f**, Substrate utilization and cell growth during EG fermentation by the strains Pp-ES (**d**), Pp-EP (**e**) and Pp∆-EP (**f**), respectively. The initial EG concentration was 56.2

mM. **g**-**i**, Substrate degradations and cell growth during mixed TPA and EG fermentation by the strains Pp-TES (**g**), Pp-TEP (**h**) and Pp△-TEP (**i**), respectively. **j**, The mcl-PHA yields in terms of dry cell or substrate weight of various production strains. The initial concentrations of TPA and EG were both 56.2 mM. Experimental data are presented as mean values with standard deviations from three independent experiments. Source data are provided as a Source Data file.

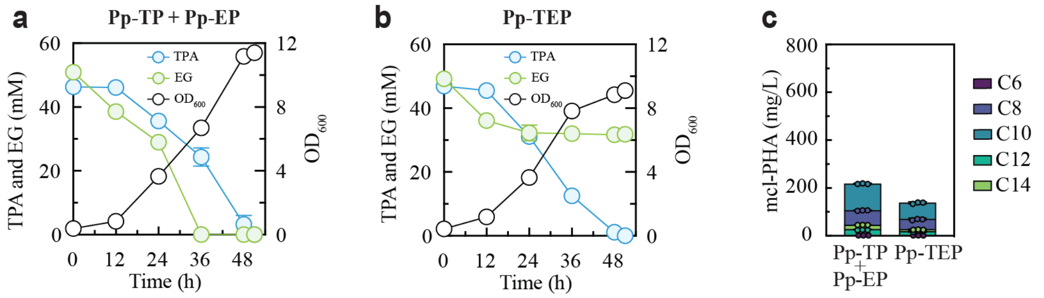

d

	Pp-TP+Pp-EP			Pp-TEP		
	**Nitrogen surplus Batch**	**Nitrogen limiting Batch**	**Nitrogen limiting Fed-batch**	**Nitrogen surplus Batch**	**Nitrogen limiting Batch**	**Nitrogen limiting Fed-batch**
**Yield (mg_{PHA}/g_{CDW})**	79.29±2.60	101.55±2.95	116.02±1.31	52.03±1.24	59.79±2.88	68.25±2.37

Supplementary Figure 9: Hydrolyzed PET fermentation for mcl-PHA production under nitrogen-surplus conditions. a,**b**, Temporal profiles of direct PET hydrolysate fermentations under nitrogen-surplus condition by the TP-EP consortium (**a**) and the single-strain counterpart Pp-TEP (**b**). An initial 1:3 inoculation ratio was used for Pp-TP and Pp-EP. **c**, The titer and monomer composition of mcl-PHA produced by the TP-EP consortium and Pp-TEP in batch fermentation under nitrogen-surplus conditions. **d**, The mcl-PHA yields from dry cell biomass through the fermentations by the TP-EP consortium and Pp-TEP in various settings. In panels **c**,**d**, mcl-productions were calculated at the time point when TPA and/or EG were fully degraded. Experimental data are presented as mean values with standard deviations from three independent experiments. Source data are provided as a Source Data file.

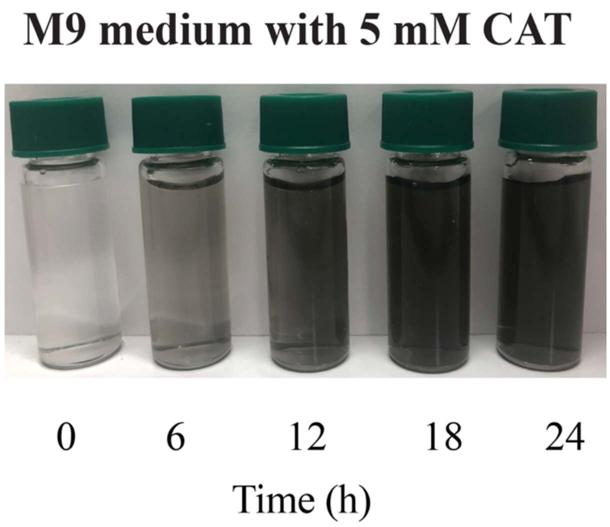

Supplementary Figure 10: Images of M9 medium containing CAT over time.

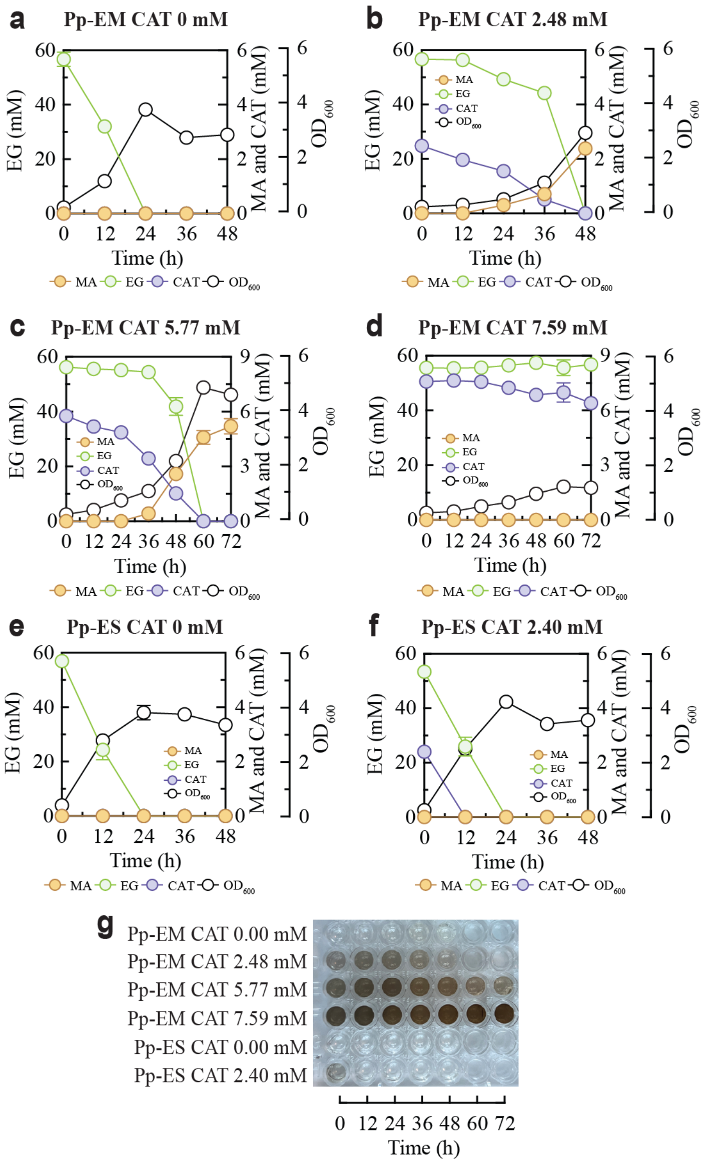

Supplementary Figure 11: CAT-to-MA conversion by Pp-EM using EG as the substrate. a-**d**, Temporal fermentation and CAT-to-MA conversion profiles of Pp-EM for 56.2 mM EG mixed with 0 mM (**a**), 2.5 mM (**b**), 5.0 mM (**c**) and 7.5 mM (**d**) of CAT**. e**,**f**, Temporal fermentation and CAT-to-MA conversion profiles of Pp-ES with 56.2 mM EG and 0 mM (**e**) or 2.5 mM (**f**) of CAT. **g**, Color change of the Pp-EM or Pp-ES culture throughout the fermentations in panels **a**-**f.** In panels **b**-**d**, the measured OD_{600} was not accurate due to the precipitation of brown carbon. Experimental data are presented as mean values with standard deviations from three independent experiments. Source data are provided as a Source Data file.

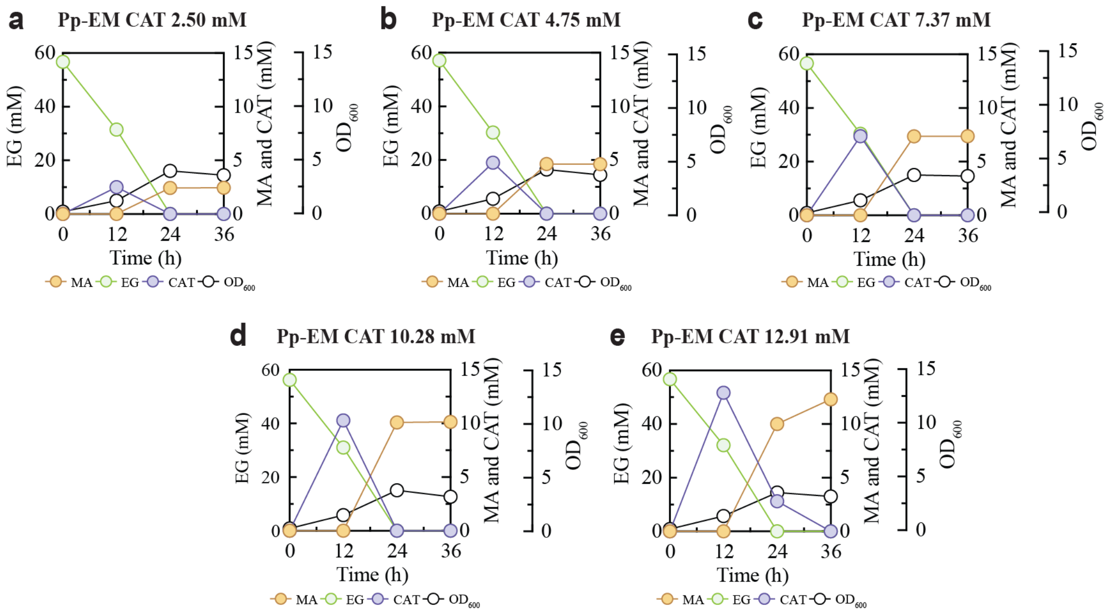

Supplementary Figure 12: MA production by Pp-EM with CAT added after 12 h of fermentation. The fermentations started with 56.2 mM EG at 0 h but were supplemented at 12 h with various levels of CAT, including 2.5 mM (**a**), 5.0 mM (**b**), 7.5 mM (**c**), 10.0 mM (**d**) and 12.5 mM (**e**). The temporal profiles of EG, CAT, MA and OD_{600} during the fermentations are shown. Experimental data are presented as mean values with standard deviations from three independent experiments. Source data are provided as a Source Data file.

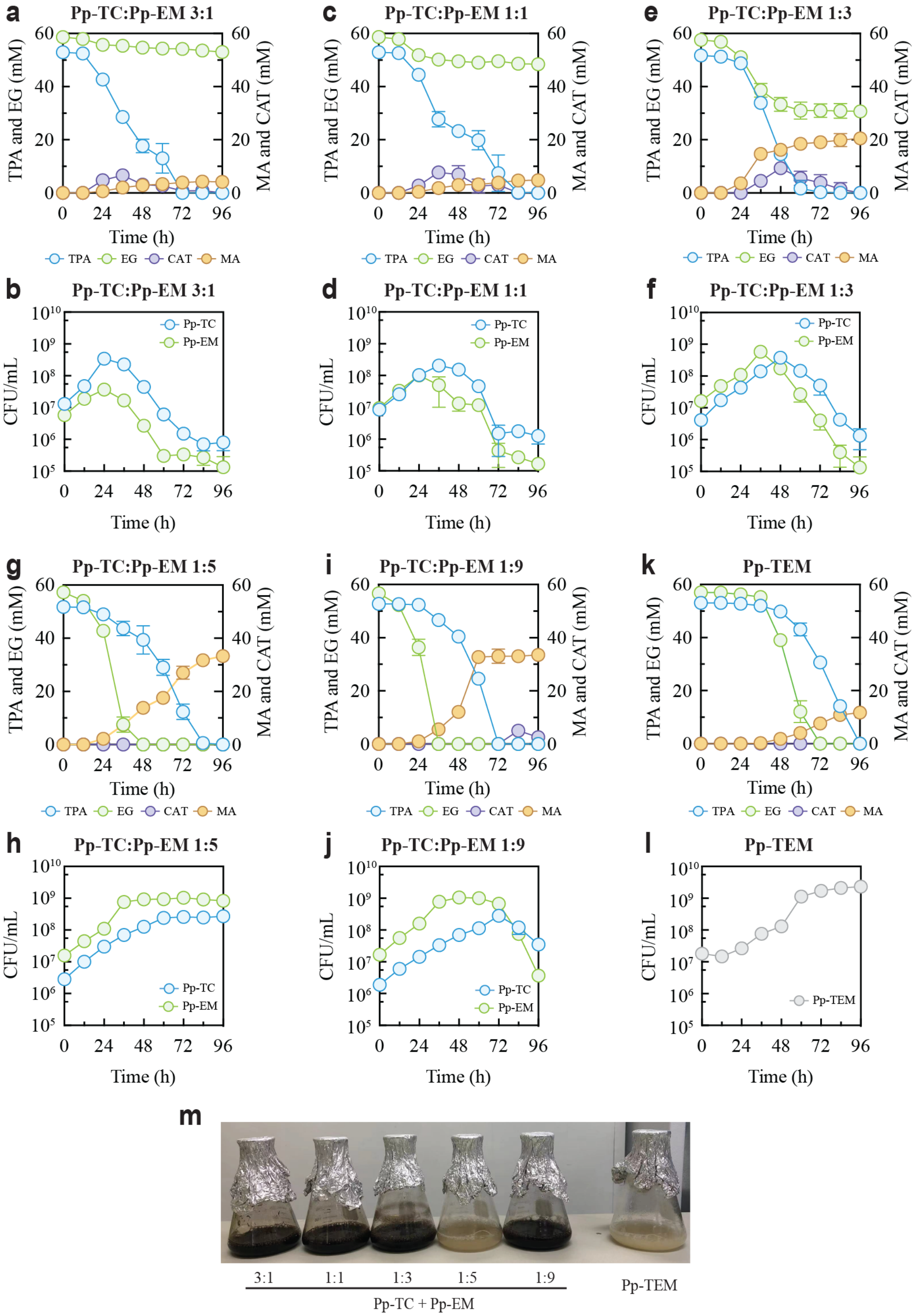

Supplementary Fig. 13: MA production from PET hydrolysate by the TC-EM consortium by altering the initial population ratio. a-j, Temporal profiles of TPA, EG, CAT, MA, and Pp-TC and Pp-EM populations during the PET hydrolysate fermentation by the TC-EM consortium. Different initial Pp-TC:Pp-EM ratios were tested, including 3:1 (**a,b**), 1:1 (**c,d**), 1:3 (**e,f**), 1:5 (**g,h**), and 1:9

(**i,j**). **k,l**, Temporal profiles of TPA, EG, CAT and MA (**k**), and Pp-TEM population (**l**) during the PET hydrolysate fermentation by the single strain Pp-TEM. **m**, Images of the fermentation broths at 96 h for the experiments in panels **a**-**l** and Fig. **5g**. Experimental data are presented as mean values with standard deviations from three independent experiments. Source data are provided as a Source Data file.

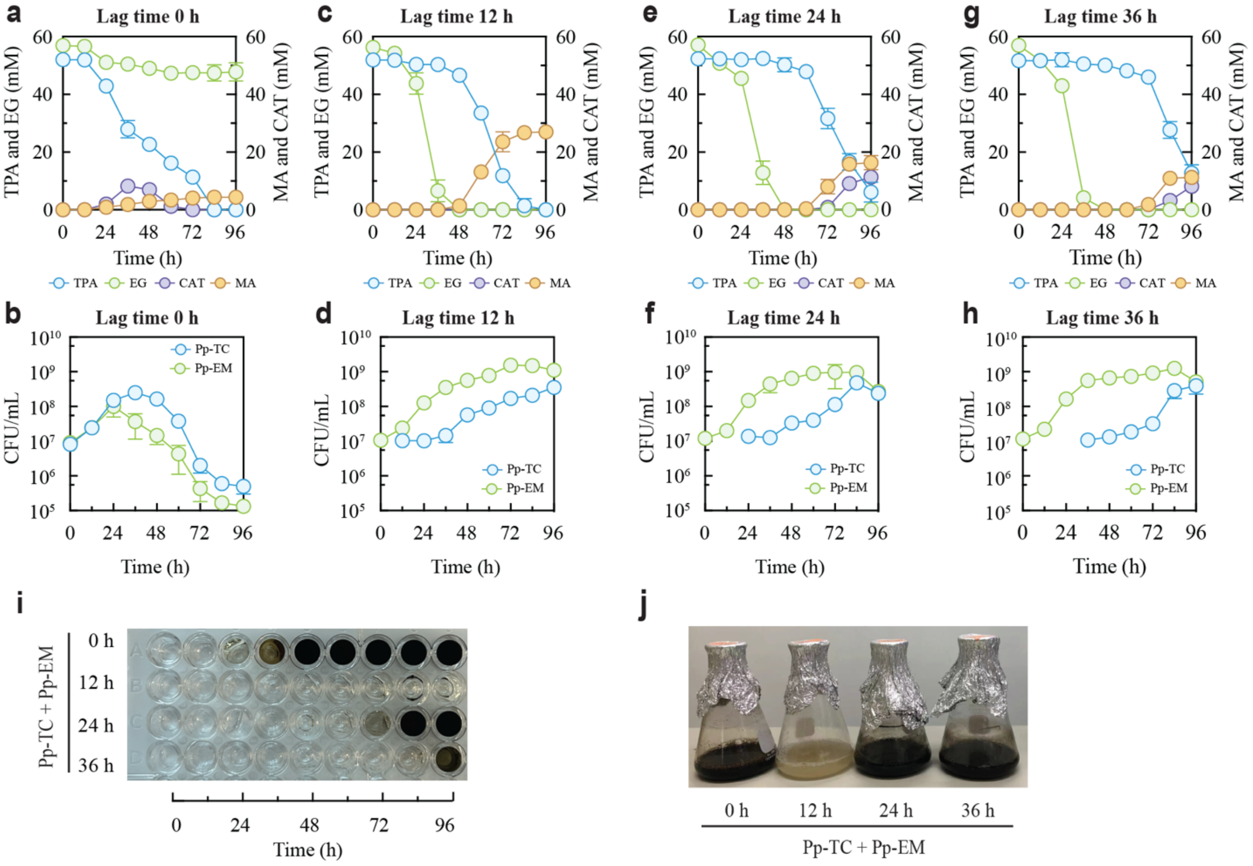

Supplementary Fig. 14: MA production from PET hydrolysate by the TC-EM consortium by varying the inoculation lag time. a-h, Temporal profiles of TPA, EG, CAT, MA, and Pp-TC and Pp-EM populations during the PET hydrolysate fermentation by the TC-EM consortium with different lag times between Pp-TC and Pp-EM inoculations. Here, Pp-TC was inoculated after Pp-EM upon different time delays, including 0 h (**a,b**), 12 h (**c,d**), 24 h (**e,f**) and 36 h (**g,h**). **i**, Color change of the TC-EM consortium culture throughout the fermentations in panels **a**-**h**. **j**, Images of the final fermentation broths at 96 h for the experiments in panels **a**-**h**. Experimental data are presented as mean values with standard deviations from three independent experiments. Source data are provided as a Source Data file.

References

1. Martínez-García, E., Nikel, P.I., Aparicio, T. & de Lorenzo, V. *Pseudomonas* 2.0: genetic upgrading of *P. putida* KT2440 as an enhanced host for heterologous gene expression. *Microbial cell factories* **13**, 1-15 (2014).
2. Cook, T.B. et al. Genetic tools for reliable gene expression and recombineering in *Pseudomonas putida*. *Journal of Industrial Microbiology & Biotechnology* **45**, 517-527 (2018).
3. Silva-Rocha, R. et al. The Standard European Vector Architecture (SEVA): a coherent platform for the analysis and deployment of complex prokaryotic phenotypes. *Nucleic Acids Research* **41**, D666-D675 (2013).
4. Schafer, A. et al. Small mobilizable multi-purpose cloning vectors derived from the *Escherichia coli* plasmids pK18 and pK19: selection of defined deletions in the chromosome of *Corynebacterium glutamicum*. *Gene* **145**, 69-73 (1994).
5. Franden, M.A. et al. Engineering *Pseudomonas putida* KT2440 for efficient ethylene glycol utilization. *Metabolic Engineering* **48**, 197-207 (2018).
6. Orellana-Saez, M. et al. In-Depth Genomic and phenotypic characterization of the antarctic psychrotolerant strain *Pseudomonas* sp. MPC6 reveals unique metabolic features, plasticity, and biotechnological potential. *Frontiers in Microbiology* **10**(2019).
7. Kenny, S.T. et al. Development of a bioprocess to convert PET derived terephthalic acid and biodiesel derived glycerol to medium chain length polyhydroxyalkanoate. *Applied Microbiology and Biotechnology* **95**, 623-633 (2012).
8. Narancic, T. et al. Genome analysis of the metabolically versatile *Pseudomonas umsongensis* GO16: the genetic basis for PET monomer upcycling into polyhydroxyalkanoates. *Microbial Biotechnology* **14**, 2463-2480 (2021).
9. Kenny, S.T. et al. Up-cycling of PET (polyethylene terephthalate) to the biodegradable plastic pha (polyhydroxyalkanoate). *Environmental Science & Technology* **42**, 7696-7701 (2008).
10. Tiso, T. et al. Towards bio-upcycling of polyethylene terephthalate. *Metabolic Engineering* **66**, 167-178 (2021).
11. Bentley, G.J. et al. Engineering glucose metabolism for enhanced muconic acid production in *Pseudomonas putida* KT2440. *Metabolic Engineering* **59**, 64-75 (2020).
12. Kohlstedt, M. et al. From lignin to nylon: Cascaded chemical and biochemical conversion using metabolically engineered *Pseudomonas putida*. *Metabolic Engineering* **47**, 279-293 (2018).
13. Liu, P. et al. Valorization of polyethylene terephthalate to muconic acid by engineering *Pseudomonas putida*. *International Journal of Molecular Sciences* **23**(2022).
14. Kohlstedt, M. et al. Biobased PET from lignin using an engineered cis, cis-muconate-producing *Pseudomonas putida* strain with superior robustness, energy and redox properties. *Metabolic Engineering* **72**, 337-352 (2022).
15. Kim, H.T. et al. Biological valorization of poly(ethylene terephthalate) monomers for upcycling waste PET. *Acs Sustainable Chemistry & Engineering* **7**, 19396-19406 (2019).
